# Supplementary material for: A multimodal characterization of low-dimensional thalamocortical structural connectivity patterns
Source: Commun Biol. 2025 Feb 5;8:185. doi: 10.1038/s42003-025-07528-8 (PMC11799188; doi:10.1038/s42003-025-07528-8)
Supplement: Supplementary file 1 — Supplementary Information [file 42003_2025_7528_MOESM1_ESM.pdf]

Supplementary Information

-

A Multimodal Characterization of Low-dimensional Thalamocortical Structural Connectivity Patterns

Alexandra John, Meike D. Hettwer, H. Lina Schaare, Amin Saberi, Şeyma Bayrak, Bin Wan, Jessica Royer, Boris C. Bernhardt, Sofie L. Valk

* Correspondence to Alexandra John (ajohn@cbs.mpg.de) and Sofie L. Valk (valk@cbs.mpg.de)

Contains:
Supplementary Figures 1 - 9
Supplementary Tables 1 – 4
Supplementary Methods

Supplementary Figures

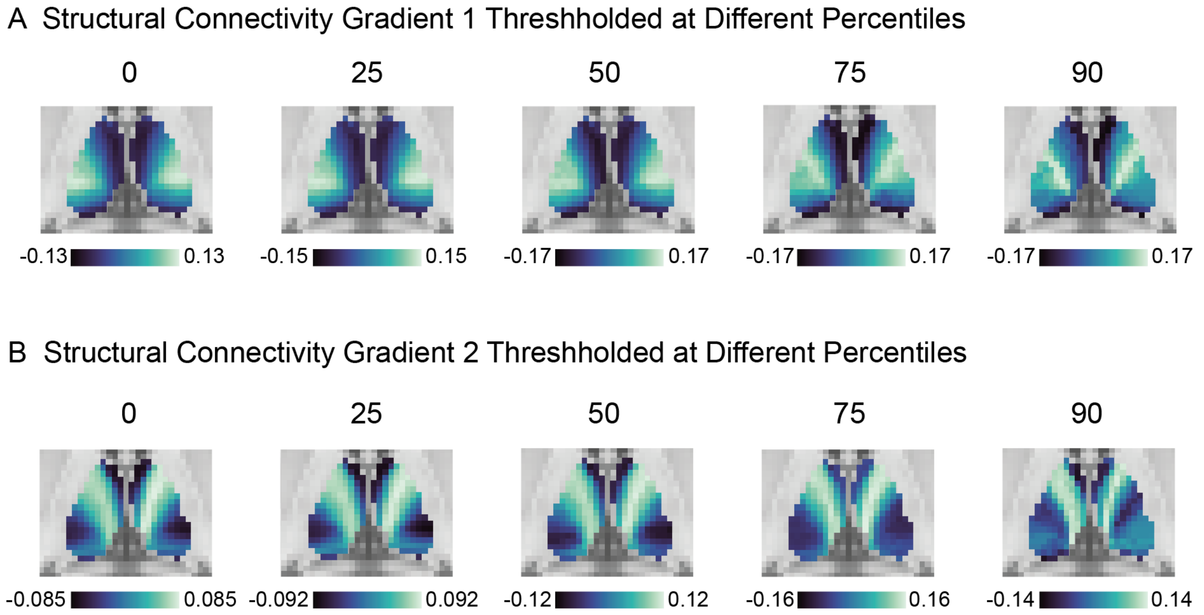

Supplementary Figure 1: Robustness of Structural Connectivity Gradients. Gradients were derived from the group-level thalamocortical structural connectivity matrix thresholded at different percentiles (0, 25, 50, 75, 90). **A** Gradient loadings of component 1 ($G1_{sc}$) were projected on the thalamus (axial plane). **B** Gradient loadings of component 2 ($G2_{sc}$) were projected on the thalamus (axial plane).

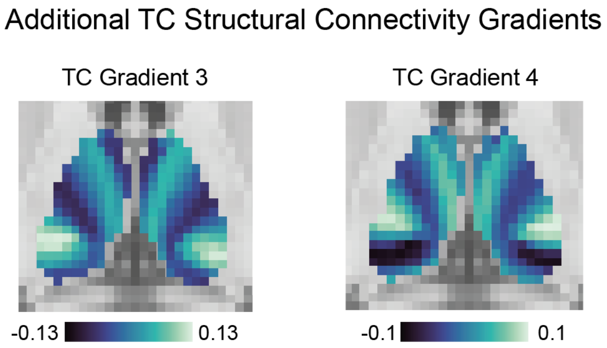

Supplementary Figure 2: Additional Thalamocortical Structural Connectivity Gradients. Gradient loadings of components 3 and 4, derived from the thalamocortical structural connectivity matrix, are projected on the thalamus (axial planes).

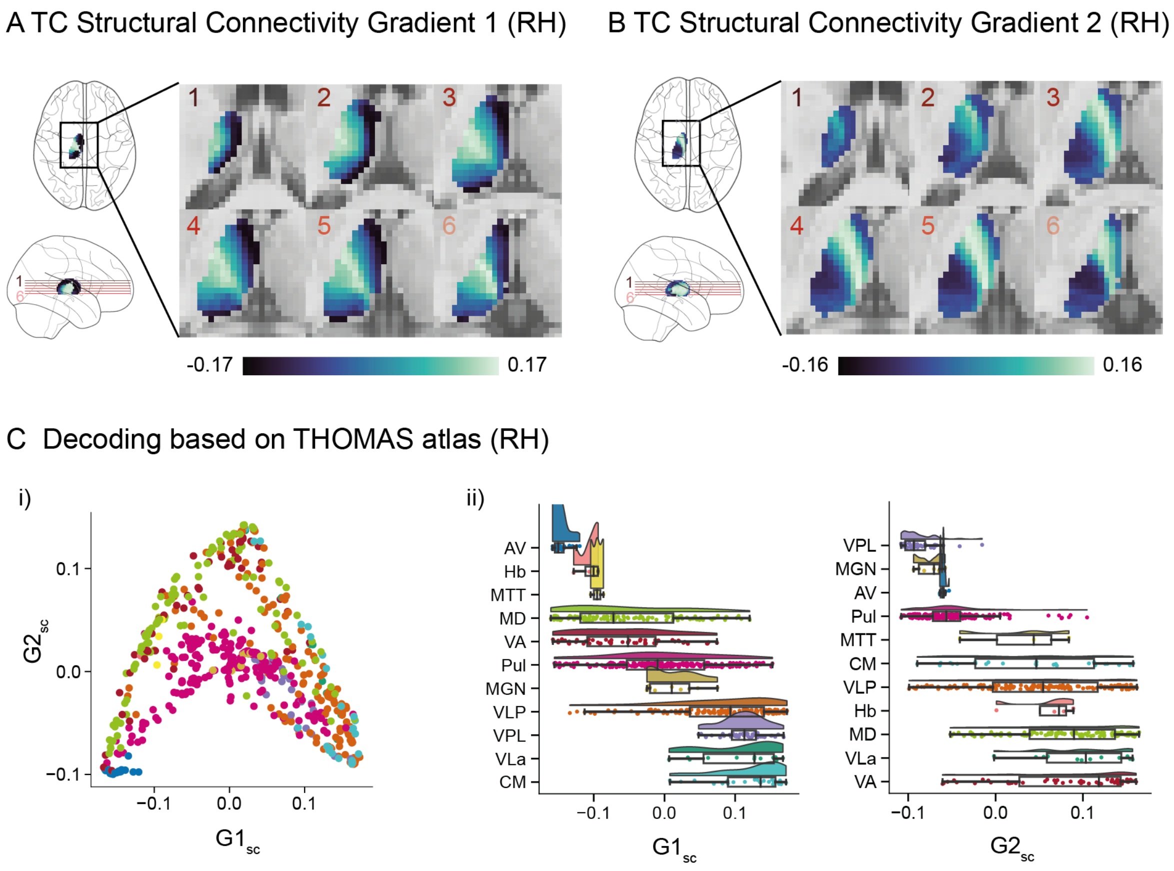

Supplementary Figure 3: Thalamocortical Structural Connectivity Gradients - Right Hemisphere (RH). A Gradient loadings of component 1 ($G1_{sc}$) projected onto the thalamus (axial planes). The red lines in the glass brain indicate the position of each respective axial slice of the displayed thalamus. **B** Gradient loadings of component 2 ($G2_{sc}$) projected on the thalamus. Slice positions are congruent to A. **C** Decoding of $G1_{sc}$ and $G2_{sc}$ based on THOMAS atlas. i) 2D space framed by $G1_{sc}$ and $G2_{sc}$, with each data point representing a thalamic voxel, color-coded by the thalamic subnucleus to which it belongs. ii) Raincloud plots display the gradient loadings of $G1_{sc}$ and $G2_{sc}$ per nucleus, ordered by median, respectively. The boxes represent the interquartile range (25th to 75th percentile), lines depict medians, and whiskers are defined by values 1.5 times the interquartile range. Abbreviations in **C**: AV: Anterior ventral nucleus, VA: Ventral anterior nucleus, VLa: Ventral lateral anterior nucleus, VLP: Ventral lateral posterior nucleus, VPL: Ventral posterior lateral nucleus, Pul: Pulvinar nucleus, MGN: Medial geniculate nucleus, CM: Centromedian nucleus, MD: Mediodorsal nucleus, Hb: Habenular nucleus, MTT: Mammillothalamic tract

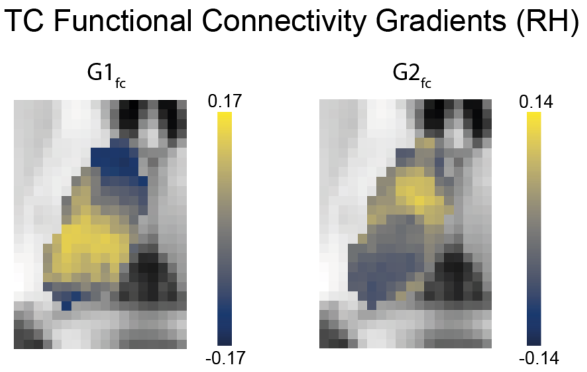

Supplementary Figure 4: Thalamocortical Functional Connectivity Gradients - Right Hemisphere (RH). RH gradient loadings of component 1 ($G1_{fc}$) and component 2 ($G2_{fc}$), derived from the thalamocortical functional connectivity matrix, projected onto the thalamus (axial slice).

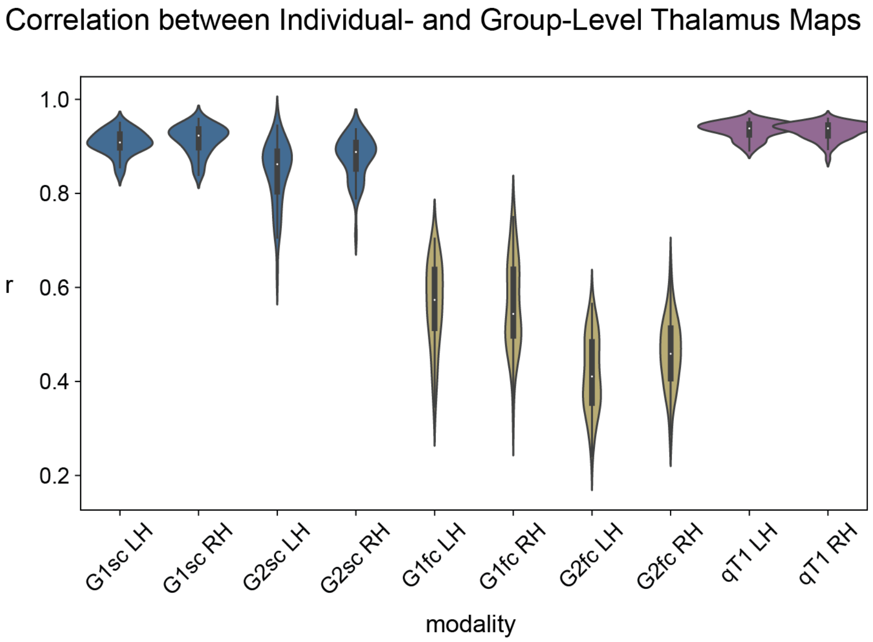

Supplementary Figure 5: Correlation Between the Individual- and Corresponding Group-Level Maps. Violin plot showing Pearson correlation (r values) between the group-level maps with the individual-level maps of each subject (N = 50) of structural connectivity gradients, functional connectivity gradients, and qT1 maps. Violin plots represent kernel density estimates of the data. The boxes indicate the interquartile range (25th to 75th percentile), the white dots represent the median, and the whiskers extend to 1.5 times the interquartile range. Abbreviations: G1sc and G2sc: structural connectivity gradient 1 and 2, G1fc and G2fc: functional connectivity gradient 1 and 2, qT1: quantitative T1 maps, LH: left hemisphere, RH: right hemisphere.

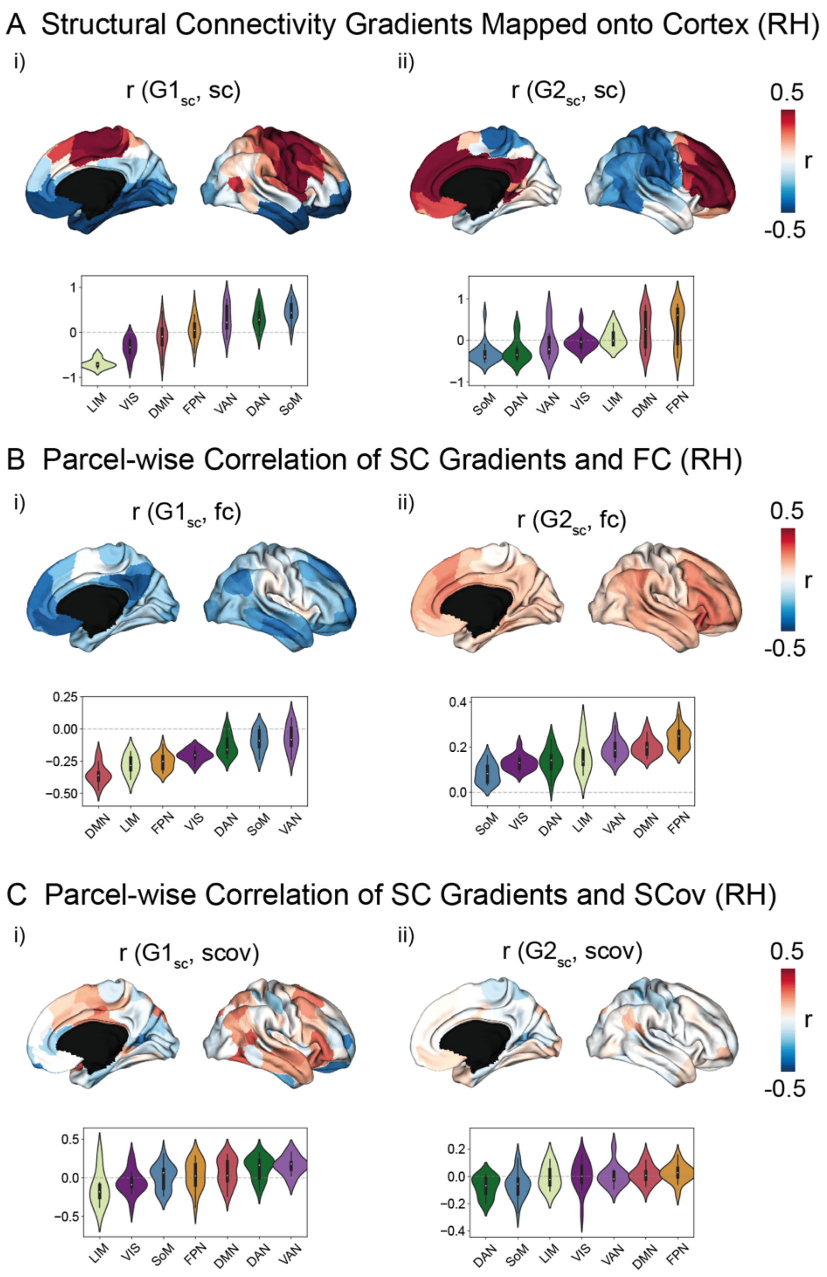

Supplementary Figure 6: Cortical Projections of Structural Connectivity Gradients and their Association with Functional Connectivity and Structural Covariance - Right Hemisphere (RH). A Structural Connectivity (sc). i) Projection of r values resulting from parcel-wise correlation between sc profiles and $G1_{sc}$ onto cortex. Thus, negative values (blue) indicate relation with the medial part of thalamus, whereas positive values (red) indicate relation with lateral thalamic portions. Decoding of cortical pattern leveraging functional communities (ordered along the mean). ii) Analog parcel-wise correlation between sc profiles and $G2_{sc}$, and decoding. **B** Functional Connectivity (fc). i) Projection of r values resulting from parcel-wise correlation between functional connectivity profiles and $G1_{sc}$ onto cortex, and decoding of cortical pattern leveraging functional communities (ordered along the mean). ii) Analog parcel-wise correlation between functional connectivity profiles and $G2_{sc}$, and decoding. **C** Structural Covariance (scov). i) Projection of r values resulting from parcel-wise correlation between structural covariance profiles and $G1_{sc}$ onto cortex, and decoding of cortical pattern leveraging functional communities (ordered along the mean). ii) Analog parcel-wise correlation between structural covariance profiles and $G2_{sc}$, and decoding. Violin plots represent kernel density estimates of the data. The boxes indicate the interquartile range (25th to 75th percentile), the white dots represent the median, and the whiskers extend to 1.5 times the interquartile range.

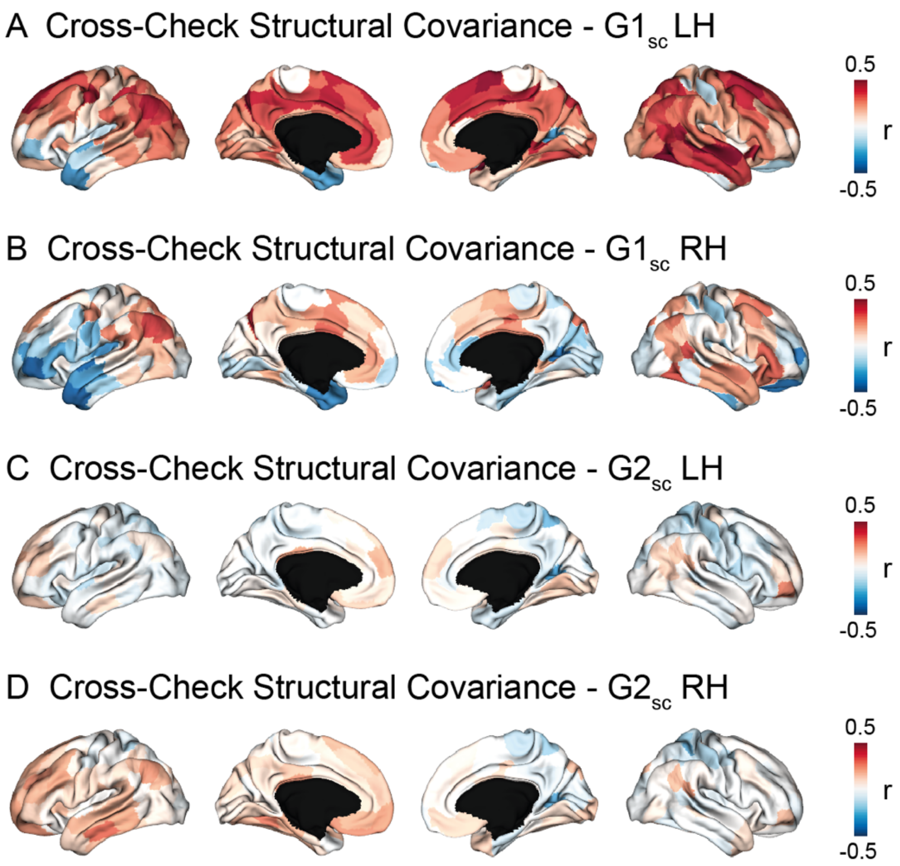

Supplementary Figure 7: Cross-Check of Structural Covariance Results. A Structural covariance was computed between left (LH) thalamic voxels to both cortical hemispheres. The resulting structural covariance profiles were parcel-wise correlated with the LH $G1_{sc}$, and r values were projected onto the cortex. **B** Structural covariance was computed between right (RH) thalamic voxels to both cortical hemispheres. The resulting structural covariance profiles were parcel-wise correlated with the RH $G1_{sc}$, and r values were projected onto the cortex. **C** Procedure analogous to A but with $G2_{sc}$. **D** Procedure analogous to B but with $G2_{sc}$.

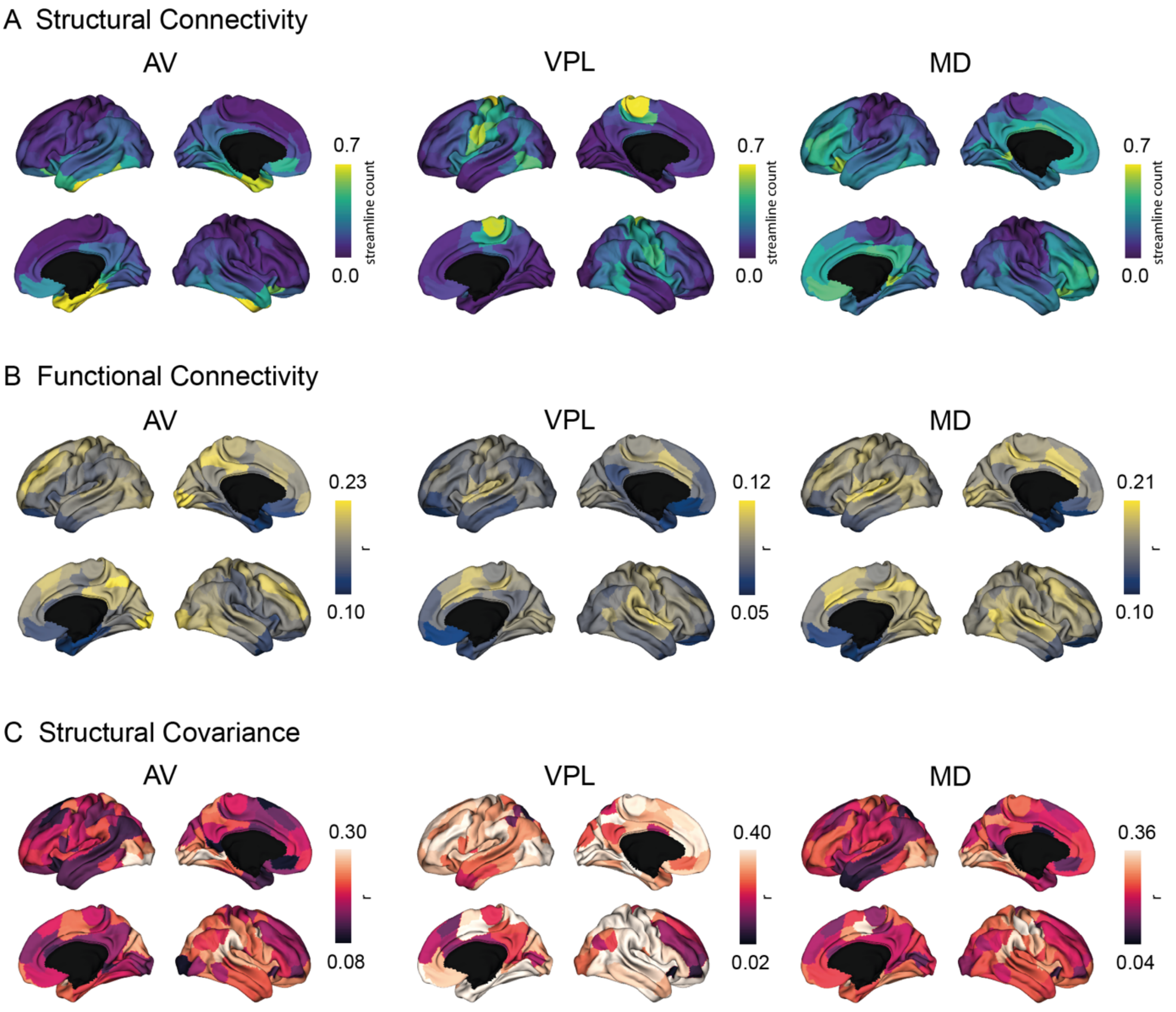

Supplementary Figure 8: Projections Based on Thomas Nuclei. A Mean structural connectivity projections (normalized streamline count) from AV, VPL, and MD projected onto the cortex. **B** Mean functional connectivity projections (r values) from AV, VPL, and MD projected onto the cortex. **C** Mean structural covariance projections (r values) from AV, VPL, and MD projected onto the cortex. Abbreviations: AV: Anterior ventral nucleus, VPL: Ventral posterior lateral nucleus, MD: Mediodorsal nucleus.

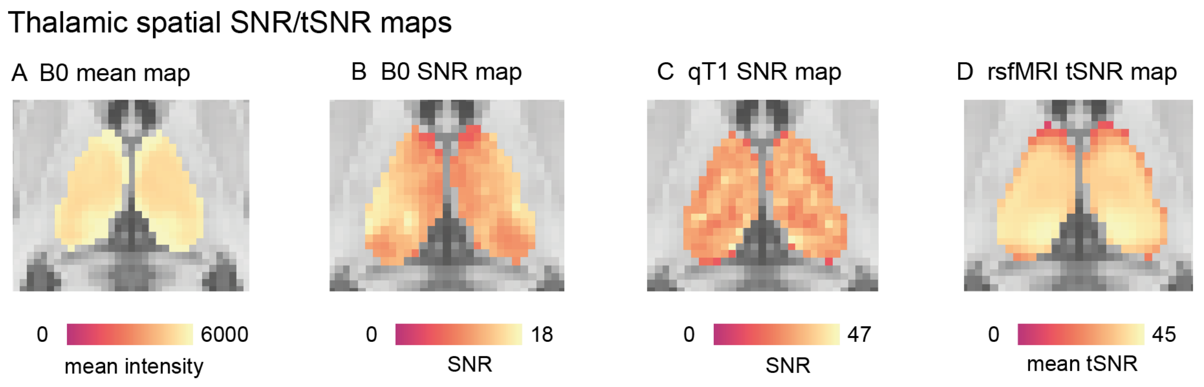

Supplementary Figure 9: Thalamic Spatial Signal-to-Noise Maps (SNR) and Temporal Signal-to-Noise Maps (tSNR). **A** Thalamic mean b0 map averaged across subjects. **B** Thalamic b0 SNR map, calculated by dividing each voxel's mean b0 intensity across subjects by its standard deviation across subjects. **C** Thalamic qT1 SNR map, calculated by dividing each voxel's mean qT1 across subjects by its standard deviation across subjects. **D** Mean rsfMRI tSNR map that was calculated by averaging individual tSNR maps. Individual tSNR maps were computed by dividing the mean time-series signal in each voxel of the motion-corrected functional data by its standard deviation.

Supplementary Tables

	group-level qT1 map	core-matrix difference map	$G1_{fc}$	$G2_{fc}$
$G1_{sc}$	LH: r = -0.536, p_{SA} = 0.038 RH: r = -0.594, p_{SA} = 0.011	LH: r = -0.378, p_{SA} = 0.135	LH: r = 0.526, p_{SA} = 0.044 RH: r = 0.564, p_{SA} = 0.014	LH: r = -0.374, p_{SA} = 0.083 RH: r = -0.133, p_{SA} = 0.532
$G2_{sc}$	LH: r = -0.068, p_{SA} = 0.873 RH: r = 0.119, p_{SA} = 0.794	LH: r = 0.676, p_{SA} = 0.044	LH: r = -0.095, p_{SA} = 0.872 RH: r = -0.177, p_{SA} = 0.754	LH: r = 0.265, p_{SA} = 0.241 RH: r = 0.484, p_{SA} = 0.016
$G3_{sc}$	LH: r = -0.112, p_{SA} = 0.411 RH: r = -0.122, p_{SA} = 0.710	LH: r = -0.017, p_{SA} = 0.949	LH: r = 0.214, p_{SA} = 0.062 RH: r = 0.290, p_{SA} = 0.157	LH: r = 0.008, p_{SA} = 0.944 RH: r = -0.039, p_{SA} = 0.806
$G4_{sc}$	LH: r = -0.132, p_{SA} = 0.461 RH: r = -0.078, p_{SA} = 0.744	LH: r = 0.233, p_{SA} = 0.098	LH: r = 0.173, p_{SA} = 0.305 RH: r = 0.079, p_{SA} = 0.740	LH: r = 0.388, p_{SA} = 0.013 RH: r = 0.367, p_{SA} = 0.018
$G5_{sc}$	LH: r = -0.010, p_{SA} = 0.967 RH: r = 0.043, p_{SA} = 0.859	LH: r = 0.093, p_{SA} = 0.615	LH: r = 0.273, p_{SA} = 0.136 RH: r = 0.223, p_{SA} = 0.245	LH: r = 0.086, p_{SA} = 0.546 RH: r = 0.082, p_{SA} = 0.592
$G6_{sc}$	LH: r = 0.027, p_{SA} = 0.917 RH: r = -0.006, p_{SA} = 0.976	LH: r = -0.193, p_{SA} = 0.394	LH: r = 0.099, p_{SA} = 0.781 RH: r = 0.028, p_{SA} = 0.894	LH: r = -0.155, p_{SA} = 0.358 RH: r = -0.106, p_{SA} = 0.384
$G7_{sc}$	LH: r = 0.129, p_{SA} = 0.242 RH: r = 0.096, p_{SA} = 0.414	LH: r = -0.068, p_{SA} = 0.356	LH: r = 0.067, p_{SA} = 0.612 RH: r = 0.002, p_{SA} = 0.987	LH: r = -0.002, p_{SA} = 0.987 RH: r = -0.024, p_{SA} = 0.822
$G8_{sc}$	LH: r = -0.063, p_{SA} = 0.759 RH: r = 0.036, p_{SA} = 0.828	LH: r = 0.081, p_{SA} = 0.622	LH: r = 0.083, p_{SA} = 0.693 RH: r = -0.020, p_{SA} = 0.927	LH: r = 0.248, p_{SA} = 0.011 RH: r = -0.085, p_{SA} = 0.320
$G9_{sc}$	LH: r = 0.047, p_{SA} = 0.528 RH: r = -0.046, p_{SA} = 0.678	LH: r = -0.004, p_{SA} = 0.941	LH: r = 0.053, p_{SA} = 0.480 RH: r = 0.033, p_{SA} = 0.789	LH: r = 0.159, p_{SA} = 0.051 RH: r = 0.103, p_{SA} = 0.345
$G10_{sc}$	LH: r = -0.136, p_{SA} = 0.417 RH: r = 0.081, p_{SA} = 0.696	LH: r = 0.324, p_{SA} = 0.020	LH: r = 0.053, p_{SA} = 0.480 RH: r = -0.095, p_{SA} = 0.662	LH: r = -0.028, p_{SA} = 0.830 RH: r = -0.175, p_{SA} = 0.251

Supplementary Table 1: Association Between TC Structural Connectivity Gradients 1 to 10 and Thalamic Maps (qT1 Group-Level Maps, Core-Matrix Map, TC Functional Connectivity Gradient 1 and 2). Pearson correlation between maps corrected for spatial autocorrelation (p_{SA}). Note that for the core-matrix map, only results for the left hemisphere are reported due to the small sample size on which the right core-matrix map is based. Abbreviations: LH: left hemisphere, RH: right hemisphere, sc: structural connectivity, fc: functional connectivity.

	group-level qT1 map	core-matrix difference map
functional connectivity gradient 1	LH: r = -0.041, p_{SA} = 0.94<br>RH: r = -0.129, p_{SA} = 0.76	LH: r = -0.211, p_{SA} = 0.76
functional connectivity gradient 2	LH: r = 0.085, p_{SA} = 0.84<br>RH: r = 0.069, p_{SA} = 0.89	LH: r = 0.568, p_{SA} = 0.01

Supplementary Table 2: Association Between TC Functional Connectivity Gradients and Intrathalamic Microstructure. Pearson correlation between functional connectivity gradients and group-level qT1 map, as well as core-atrix map, corrected for spatial autocorrelation (pSA). Note that for the core-matrix map, only results for the left hemisphere are reported due to the small sample size on which the right core-matrix map is based. Abbreviations: LH: left hemisphere, RH: right hemisphere.

	Oldham and Ball (2023)		
	main (gene expression + structural connectivity	structural connectivity	gene expression
$G1_{sc}$	LH: r = 0.478,<br>p_{SA} = 0.037	LH: r = - 0.061,<br>p_{SA} = 0.778	LH: r = 0.423,<br>p_{SA} = 0.054
$G2_{sc}$	LH: r = -0.701,<br>p_{SA} = 0.039	LH: r = - 0.817,<br>p_{SA} = < 0.000	LH: r = -0.585,<br>p_{SA} = 0.227

Supplementary Table 3: Association Between TC Structural Connectivity Gradients and Gradients from Oldham and Ball (2023). Pearson correlation between maps corrected for spatial autocorrelation (p_{SA}). Note that the analysis was conducted solely for the left hemisphere, as the data provided by Oldham and Ball (2023) is restricted to this side only. Abbreviations: LH: left hemisphere, sc: structural connectivity

	Mean b0 map	SNR B0 map	SNR qT1 map	tSNR rsfMRI map
$G1_{sc}$	LH: r = 0.390,<br>p_{SA}= 0.110<br>RH: r = -0.492,<br>p_{SA}= 0.008	LH: r = 0.717,<br>p_{SA} < 0.000<br>RH: r = 0.724,<br>p_{SA} = < 0.000	LH: r = 0.213,<br>p_{SA} = 0.197<br>RH: r = 0.171,<br>p_{SA} = 0.273	LH: r = 0.334,<br>p_{SA} = 0.192<br>RH: r = 0.245,<br>p_{SA} = 0.364
$G2_{sc}$	LH: r = -0.323,<br>p_{SA} = 0.199,<br>RH: r = -0.142,<br>p_{SA} = 0.624	LH: r = 0.1773,<br>p_{SA} = 0.567<br>RH: r = 0,167,<br>p_{SA} = 0.595	LH: r = 0.222,<br>p_{SA} = 0.184<br>RH: r = 0,099,<br>p_{SA} = 0.528	LH: r = 0.158,<br>p_{SA} = 0.581<br>RH: r = 0.160,<br>p_{SA} = 0.618

Supplementary Table 4: Association Between TC Structural Connectivity Gradients and SNR/tSNR Maps. Pearson correlation between structural connectivity gradients ($G1_{sc}$, $G2_{sc}$) and thalamic mean b0 map, SNR b0 map, SNR qT1 map, and tSNR rsfMRI map, corrected for spatial autocorrelation (p_{SA}). Abbreviations: LH: left hemisphere, sc: structural connectivity

Supplementary Methods

Robustness of structural connectivity gradients computed with different thresholds: To check the robustness of the structural connectivity gradient patterns, we computed gradients by thresholding the input group-level structural connectivity matrix at different percentiles (0, 25, 50, 75, 90) (Supplementary Figure 1).

Within-modality correlation between the individual maps and the group-level maps: To investigate consistency, we calculated structural connectivity gradients, functional connectivity gradients, and qT1 maps at the individual level. Gradients were Procrustes aligned to the group-level maps using BrainSpace (v. 0.1.3). Individual maps were then correlated (Pearson) with the corresponding group-level maps (Supplementary Figure 5).

Cross-check structural covariance: We calculated the structural covariance between the left thalamus and the cortical parcels of both hemispheres and correlated the resulting structural covariance profiles parcel-wise with the left $G1_{sc}$ and $G2_{sc}$. We did the same analysis steps for the right hemisphere, respectively (Supplementary Figure 7).

Projections based on THOMAS atlas: Using the THOMAS atlas in MNI space, we calculated the mean projections of the nuclei AV, VPL, and MD for structural connections, functional connectivity, and structural covariance (Supplementary Figure 8).

Association between functional connectivity gradients and thalamic microstructure: To additionally investigate associations between thalamic functional connectivity gradients and thalamic microstructure, we calculated Pearson correlation between the first two functional connectivity gradients and the group-level qT1 map and core-matrix map (Supplementary Table 2).

Association between structural connectivity gradients and thalamic gradients from Oldham and Ball (2023): To further test the generalizability of our findings, we compared our gradients to those reported by Oldham and Ball (2023), who derived thalamic gradients based on transcriptomic and structural connectivity data. Their source data is available at https://doi.org/10.1038/s41467-023-41722-8. $G1_{sc}$ and $G2_{sc}$ from our study where Pearson correlated with Oldham and Ball's main thalamic gradient based on gene expression data joint with structural connectivity (Oldham and Ball, 2023, Fig 2a), and their gradients separately based on gene expression (Oldham and Ball, 2023, Fig S3b), and structural connectivity (Oldham and Ball, 2023, Fig S3c) (Supplementary Table 3).

Thalamic SNR and temporal SNR maps and their association to structural connectivity gradients: We investigated the spatial distribution of SNR and temporal SNR (tSNR) in the thalamus. For DWI, the mean of each subject's b0 images was computed and warped to MNI space. We show the spatial SNR maps, calculated by dividing each voxel's mean across subjects by each voxel's standard deviation across subjects. This was analog used for the calculation of qT1 spatial SNR maps. For rsfMRI data, tSNR was computed per subject by dividing the mean

time-series signal in each voxel of the motion-corrected functional data by its standard deviation, followed by warping the subject's tSNR maps to MNI space and calculating the mean across subjects resulting in a group-level spatial tSNR map (Supplementary Figure 9). Following, we evaluated the spatial association between SNR/tSNR and thalamic gradients by correlating (Pearson) the maps with $G1_{sc}$ and $G2_{sc}$ and corrected for spatial autocorrelation (Supplementary Table 4).
