# Supplementary material for: A multimodal characterization of low-dimensional thalamocortical structural connectivity patterns
Source: Commun Biol. 2025 Feb 5;8:185. doi: 10.1038/s42003-025-07528-8 (PMC11799188; doi:10.1038/s42003-025-07528-8)
Supplement: Supplementary file 2 — Description of Additional Supplementary Materials [file 42003_2025_7528_MOESM2_ESM.pdf]

Description of Additional Supplementary Files

File name: Supplementary Data 1

Description: . Source data behind the main figures
