# Supplementary material for: A multimodal characterization of low-dimensional thalamocortical structural connectivity patterns
Source: Commun Biol. 2025 Feb 5;8:185. doi: 10.1038/s42003-025-07528-8 (PMC11799188; doi:10.1038/s42003-025-07528-8)
Supplement: Supplementary file 4 — Reporting Summary [file 42003_2025_7528_MOESM4_ESM.pdf]

Corresponding author(s): Alexandra John, Dr. Sofie L. Valk

Last updated by author(s): 07/01/2025

Reporting Summary

Nature Portfolio wishes to improve the reproducibility of the work that we publish. This form provides structure for consistency and transparency in reporting. For further information on Nature Portfolio policies, see our Editorial Policies and the Editorial Policy Checklist.

Statistics

For all statistical analyses, confirm that the following items are present in the figure legend, table legend, main text, or Methods section.

n/a	Confirmed	
☐	☒	The exact sample size (*n*) for each experimental group/condition, given as a discrete number and unit of measurement
☒	☐	A statement on whether measurements were taken from distinct samples or whether the same sample was measured repeatedly
☐	☒	The statistical test(s) used AND whether they are one- or two-sided<br>*Only common tests should be described solely by name; describe more complex techniques in the Methods section.*
☒	☐	A description of all covariates tested
☐	☒	A description of any assumptions or corrections, such as tests of normality and adjustment for multiple comparisons
☒	☐	A full description of the statistical parameters including central tendency (e.g. means) or other basic estimates (e.g. regression coefficient) AND variation (e.g. standard deviation) or associated estimates of uncertainty (e.g. confidence intervals)
☒	☐	For null hypothesis testing, the test statistic (e.g. F, t, r) with confidence intervals, effect sizes, degrees of freedom and P value noted<br>*Give P values as exact values whenever suitable.*
☒	☐	For Bayesian analysis, information on the choice of priors and Markov chain Monte Carlo settings
☒	☐	For hierarchical and complex designs, identification of the appropriate level for tests and full reporting of outcomes
☐	☒	Estimates of effect sizes (e.g. Cohen's *d*, Pearson's *r*), indicating how they were calculated

Our web collection on statistics for biologists contains articles on many of the points above.

Software and code

Policy information about availability of computer code

Data collection	The data used in this study was obtained from the openly available multimodal MRI dataset for Microstructure-Informed Connectomics (MICA-MICs; Royer et al., 2022). Please see our Data Availibility Statement.
Data analysis	All code can be found at the Github Repository: https://github.com/CNG-LAB/cngopen/tree/main/thalamic_gradients. Used open Software is mentioned in the manuscript.

For manuscripts utilizing custom algorithms or software that are central to the research but not yet described in published literature, software must be made available to editors and reviewers. We strongly encourage code deposition in a community repository (e.g. GitHub). See the Nature Portfolio guidelines for submitting code & software for further information.

Data

Policy information about availability of data

All manuscripts must include a data availability statement. This statement should provide the following information, where applicable:

- Accession codes, unique identifiers, or web links for publicly available datasets
- A description of any restrictions on data availability
- For clinical datasets or third party data, please ensure that the statement adheres to our policy

The dataset used in this study is openly available and can be downloaded from: CONP Portal (https://portal.conp.ca/dataset?id=projects/mica-mics) and OSF (https://osf.io/j532r/ with the identifier doi 10.17605/OSF.IO/J532R)91,92. As a basis for our thalamus mask, we used the openly accessible Harvard-Oxford

subcortical atlas, integrated in FSL (https://ftp.nmr.mgh.harvard.edu/pub/dist/freesurfer/tutorial_packages/centos6/fsl_507/doc/wiki/Atlases.html). The template of the THOMAS atlas, which was used to identify thalamic nuclei, can be requested here: https://doi.org/10.5281/zenodo.549950458. The core-matrix difference map can be downloaded from: https://github.com/macshine/corematrix60. For supplementary analysis, we compared our structural connectivity gradients with those from Oldham and Ball, 202343; their source data is available here: https://doi.org/10.1038/s41467-023-41722-8. Data generated in this study, such as group-level structural connectivity matrix, group-level functional connectivity matrix, group-level structural covariance, and group-level qT1 map have been deposited on Github (https://github.com/CNG-LAB/cngopen/tree/main/thalamic_gradients). Source data behind the main figures can be found in Supplementary Data 1.

Research involving human participants, their data, or biological material

Policy information about studies with human participants or human data. See also policy information about sex, gender (identity/presentation), and sexual orientation and race, ethnicity and racism.

Reporting on sex and gender	The dataset used in this study was acquired from 50 healthy adults (sex: 23 female and 27 male).
Reporting on race, ethnicity, or other socially relevant groupings	na
Population characteristics	age mean ± SD = 29.54 ± 5.62 years
Recruitment	na
Ethics oversight	The original study (Royer et al., 2022, https://doi.org/10.1038/s41597-022-01682-y) was approved by the ethics committee of the Montreal Neurological Institute and Hospital.

Note that full information on the approval of the study protocol must also be provided in the manuscript.

Field-specific reporting

Please select the one below that is the best fit for your research. If you are not sure, read the appropriate sections before making your selection.

☒ Life sciences ☐ Behavioural & social sciences ☐ Ecological, evolutionary & environmental sciences

For a reference copy of the document with all sections, see nature.com/documents/nr-reporting-summary-flat.pdf

Life sciences study design

All studies must disclose on these points even when the disclosure is negative.

Sample size	The data sample (MICA-MICs) consisted of 50 (23 female) individuals with age mean ± SD = 29.54 ± 5.62 years.
Data exclusions	After quality control, we did not exclude data from the dataset.
Replication	We did not replicate our experiments beyond evaluating consistency between left and right thalamus, testing consistency between individual and group-level maps, comparing to previous findings (Oldham and Ball, 2023), and performing multimodal analysis.
Randomization	We evaluated mean profiles of structural and functional organization of the thalamus. Hence we did not have experimental conditions.
Blinding	Blinding was not relevant for the current study, focusing on spatial variability of anatomy and intrinsic function within the thalamus and between thalamus and cortex.

Reporting for specific materials, systems and methods

We require information from authors about some types of materials, experimental systems and methods used in many studies. Here, indicate whether each material, system or method listed is relevant to your study. If you are not sure if a list item applies to your research, read the appropriate section before selecting a response.

Materials & experimental systems

n/a	Involved in the study	
☒	☐	Antibodies
☒	☐	Eukaryotic cell lines
☒	☐	Palaeontology and archaeology
☒	☐	Animals and other organisms
☒	☐	Clinical data
☒	☐	Dual use research of concern
☒	☐	Plants

Methods

n/a	Involved in the study	
☒	☐	ChIP-seq
☒	☐	Flow cytometry
☐	☒	MRI-based neuroimaging

Plants

Seed stocks: -

Novel plant genotypes: -

Authentication: -

Magnetic resonance imaging

Experimental design

Design type: Resting state functional MRI, quantitative MRI, and diffusion MRI

Design specifications: Royer et al., 2022: Participants underwent a T1-weighted (T1w) structural scan, followed by multi-shell diffusion-weighted imaging (DWI) and resting-state functional MRI (rs-fMRI). In addition, a pair of spin-echo images was acquired for distortion correction of individual rs-fMRI scans. A second T1w scan was then acquired, followed by qT1 mapping. Total scan time for these acquisitions was approximately 45 minutes.
We used standard resting-state parameters, averaged overe time, please see below.

Behavioral performance measures: We did not evaluate behavioral performance in the current study.

Acquisition

Imaging type(s): structural (T1W, qT1), functional (rsfMRI), diffusion

Field strength: 3 T

Sequence & imaging parameters: T1w: Using a 3D magnetization-prepared rapid gradient-echo sequence (MP-RAGE), two T1w images with identical parameters (0.8 mm isotropic voxels, matrix = 320 × 320, 224 sagittal slices, TR = 2300 ms, TE = 3.14 ms, TI = 900 ms, flip angle = 9°, iPAT = 2, partial Fourier = 6/8) were acquired.
rsFMRI: Resting-state fMRI images were acquired during a 7 min scan session using multiband accelerated 2D-BOLD echo-planar imaging (3 mm isotropic voxels, TR = 600 ms, TE = 30 ms, flip angle = 52°, FOV = 240 × 240 mm2, slice thickness = 3 mm, mb factor = 6, echo spacing = 0.54 ms). For distortion correction of fMRI scans two spin-echo images with reverse phase encoding (3 mm isotropic voxels, TR = 4029 ms, TE = 48ms, flip angle = 90°, FOV = 240 × 240 mm2, slice thickness = 3mm, echo spacing = 0.54 ms, phase encoding = AP/PA, bandwidth = 2084 Hz/Px) were acquired. Participants HC001 to HC004 underwent slightly longer acquisition (800 time points) but for consistency, we use the same number of time points for all subjects (700 time points).
qT1: The qT1 relaxometry data were acquired using a 3D magnetization prepared 2 rapid acquisition gradient echoes sequence (MP2RAGE; 0.8 mm isotropic voxels, 240 sagittal slices, TR = 5000 ms, TE = 2.9 ms, TI 1 = 940 ms, T1 2 = 2830 ms, flip angle 1 = 4°, flip angle 2 = 5°, iPAT = 3, bandwidth = 270 Hz/px, echo spacing = 7.2 ms, partial Fourier = 6/8). To reduce sensitivity to B1 inhomogeneities and to optimize intra- and inter-subject reliability, two inversion images were combined for qT1 mapping.

Area of acquisition: whole brain scan

Diffusion MRI: ☒ Used ☐ Not used

Parameters: For the acquisition of the multi-shell DWI data a spin-echo echo-planar imaging sequence was used. This sequence consists of three shells with b-values 300, 700, and 2000s/mm2 and 10, 40, and 90 diffusion weighting directions per shell, respectively (1.6 mm

isotropic voxels, TR = 3500 ms, TE = 64.40 ms, flip angle = 90°, refocusing flip angle = 180°, FOV = 224 × 224 mm2, slice thickness = 1.6 mm, multi-band factor = 3, echo spacing = 0.76 ms, number of b0 images = 3). Additionally, b0 images in reverse phase encoding directions are provided for distortion correction of DWI scans.

Preprocessing

Preprocessing software

For the preprocessing of MRI data, we used the specific modules of the containerized multimodal MRI processing tool micapipe (v. 0.1.2; Cruces et al., 2022) to ensure robustness and reproducibility.

Normalization

We normalized the data to be aligned to MNI space using non-linear and linear approaches as described in Royer et al., 2022

Normalization template

MNI152 template

Noise and artifact removal

Based on Royer, 2022:
DWI pre-processing: DWI data were pre-processed using MRtrix. DWI data was denoised, underwent b0 intensity normalization, and were corrected for susceptibility distortion, head motion, and eddy currents using a reverse phase encoding from two b=0s/mm2 volumes

rs-fMRI pre-processing rs-fMRI images were pre-processed using AFNI and FSL. The first five volumes were discarded to ensure magnetic field saturation. Images were reoriented, as well as motion and distortion corrected. Motion correction was performed by registering all timepoint volumes to the mean volume, while distortion correction leveraged main phase and reverse phase field maps acquired alongside rs-fMRI scans. Nuisance variable signal was removed using an ICA-FIX classifier trained in-house on a subset of 30 participants (15 healthy controls, 15 drug-resistant epilepsy patients) and by performing spike regression using motion outlier outputs provided by FSL. Volumetric timeseries were averaged for registration to native FreeSurfer space using boundary-based registration, and mapped to individual surface models using trilinear interpolation.

Volume censoring

Please see above.

Statistical modeling & inference

Model type and settings

We studied spatial correlations between measures.

Effect(s) tested

See above

Specify type of analysis: ☐ Whole brain ☒ ROI-based ☐ Both

Anatomical location(s): thalamus, cortex (schaefer 200 parcellation)

Statistic type for inference
(See Eklund et al. 2016)

Correlation (Pearson)

Correction

We used variograms (brainsmash toolbox, v. 0.11.0; https://brainsmash.readthedocs.io/en/latest/) for spatial autocorrelation correction.

Models & analysis

n/a	Involved in the study
☐	☒ Functional and/or effective connectivity
☒	☐ Graph analysis
☒	☐ Multivariate modeling or predictive analysis

Functional and/or effective connectivity

Pearson correlation and Fisher's z transform

nature portfolio | reporting summary
